# Antipsychotic and Psychotropic Prescribing in Assisted Living

**DOI:** 10.1093/geroni/igaa057.2481

**Published:** 2020-12-16

**Authors:** Sheryl Zimmerman, Philip Sloane, Christopher Wretman, Stephanie Miller, John Preisser

**Affiliations:** 1 University of North Carolina at Chapel Hill, Chapel Hill, North Carolina, United States; 2 Cecil G. Sheps Center for Health Services Research, Chapel Hill, North Carolina, United States

## Abstract

The Center for Medicare & Medicaid Services has brought national attention to potentially inappropriate antipsychotic prescribing for residents with dementia in nursing homes, but no such attention has been paid to prescribing in assisted living (AL), despite the fact that more than 40% of AL residents have moderate/severe dementia. This study examined antipsychotic and other psychotropic prescribing in AL using a seven-state sample of 250 communities, and examined correlates of prescribing; analyses used weights based on probability proportional to bed size to derive state-level estimates. Overall, 19% of residents were taking an antipsychotic medication (27% of those with dementia); corresponding numbers for antidepressants were 46% (54%) and for anxiolytics/hypnotics were 24% (28%; both p <.001). By way of example, communities that did not have an RN or LPN were more likely to prescribe antipsychotics (34% vs. 17%; p<.001). The discussion will highlight next steps regarding practice, policy, and research.

